# Xylitol promotes the antioxidant and biocontrol efficiency of the antagonistic yeast, *Meyerozyma guilliermondii*

**DOI:** 10.3389/fmicb.2025.1545248

**Published:** 2025-05-09

**Authors:** Weiwei Zhang, Shunyao Zhuang, Jianshuang Gao

**Affiliations:** ^1^State Key Laboratory of Soil and Sustainable Agriculture, Institute of Soil Science, Chinese Academy of Sciences, Nanjing, China; ^2^University of the Chinese Academy of Sciences, Beijing, China; ^3^School of Economic Geography, Hunan University of Finance and Economics, Changsha, China

**Keywords:** *Meyerozyma guilliermondii*, biocontrol efficiency, xylitol, antagonist, gray mold

## Abstract

The biocontrol efficiency of the antagonist yeast *Meyerozyma guilliermondii* is significantly reduced under oxidative stress in adverse environments. However, effective strategies to improve *M. guilliermondii* under such abiotic stress remain limited. As an effective protectant of yeasts, xylitol has significant potential to improve the performance of *M. guilliermondii* under abiotic stress. We investigated xylitol’s effects on the viability and efficiency of *M. guilliermondii* under oxidative stress. The results showed that 0.5 M and 1 M xylitol significantly enhanced yeast survival, antioxidant gene expression, and enzyme activity, including thioredoxin reductase (TrxR) and peroxidase (POD), while reducing intracellular reactive oxygen species levels as well as damage to mitochondrial membranes, and preserving the ATP content. Notably, xylitol-treated (XT) yeast exhibited higher intracellular xylitol levels and improved resistance to oxidative stress compared with the non-xylitol-treated cells. Additionally, XT yeast showed a greater biocontrol efficacy and lower postharvest fungal infection rate by gray mold and blue mold in apples. These results demonstrated that xylitol effectively boosts the resilience and biocontrol efficiency of *M. guilliermondii*, making it a promising candidate to improve postharvest disease management.

## 1 Introduction

Postharvest fungal control using biocontrol yeasts offers both practical and economic advantages (Droby et al., 2016; Wisniewski et al., 2016; Kim et al., 2018). Over recent decades, a number of biocontrol yeast strains have been identified as effective antagonists against fungal diseases, such as gray mold (*Botrytis cinerea*) and blue mold (*Penicillium expansum*) (Romanazzi et al., 2016; Vilanova et al., 2017). Among the effective biocontrol yeasts, *Meyerozyma guilliermondii* shows high efficacy against various postharvest fungal pathogens of multiple fruits, including apples, pears, and kiwifruit during storage (Sui and Liu, 2014; Sadeghi et al., 2021).

Despite the demonstrated efficacy of antagonistic yeasts, their efficacy as biocontrol agents can be constrained by environmental factors, such as temperature, oxidative stress, and salinity, thereby limiting their effectiveness (Macarisin et al., 2010; Sui et al., 2015; Spadaro and Droby, 2016). The stress tolerance of antagonistic yeasts is closely linked to their survival and proliferation within host tissues, as well as their biocontrol efficacy against pathogens (Liu et al., 2013). Consequently, identifying effective and cost-efficient protectants is a strategic approach to enhance the biocontrol capacity of these yeasts, offering both efficiency and rapid benefits (Liu et al., 2011; Ming et al., 2020).

Sugars or sugar alcohols have been shown to be effective protectants for biocontrol yeasts (Sui et al., 2012; Ming et al., 2020). Xylitol, a polyol derived from the hydrogenation of xylose, has demonstrated efficacy against ochratoxigenic fungus *Aspergillus carbonarius* and *Listeria monocytogenes* (Morón de Salim and Ramírez Mérida, 2013; Espinosa-Salgado et al., 2022). Xylitol’s low cost and widespread use as a food additive, along with its inherent antioxidant properties, make it particularly well-suited for use as a protectant for biocontrol yeasts in both pre-and post-harvest applications. However, there is limited research on the application of these compounds as protectants for antagonistic yeasts under adverse environmental conditions.

Taking into account the above research, the present study was conducted to determine the effects of xylitol on the antioxidant response, stress tolerance, and biocontrol efficacy of the yeast *M. guilliermondii*. We determined: (1) The cell survival rate of xylitol-treated (XT) cells following exposure to oxidative stress induced by H_2_O_2_; (2) the impact of xylitol treatment on the expression of antioxidant genes, including those encoding thioredoxin reductase (TrxR) and peroxidase (POD), as well as their corresponding enzyme activities; (3) the accumulation of intracellular reactive oxygen species (ROS), mitochondrial membrane potential analysis, changes in ATP production and intracellular xylitol levels; and (4) the biocontrol efficacy of XT *M. guilliermondii* yeast cells against *B. cinerea* and *P. expansum* infections in apples.

## 2 Materials and methods

### 2.1 Antagonistic yeast

The yeast *M. guilliermondii*, known for its antagonistic properties, was first isolated from apple surfaces and identified through its general morphology and DNA sequencing of the ribosomal internal transcribed spacer (ITS) region (Leaw et al., 2006). This yeast was cultured in a yeast extract-peptone-dextrose (YPD) medium, which contained 10 g of yeast extract, 20 g of peptone, and 20 g of glucose dissolved in 1 liter of water. To cultivate the yeast, 40 mL of YPD medium was added to a 100 mL Erlenmeyer flask, followed by inoculation with *M. guilliermondii*. The initial yeast cell concentration was measured at 10^5^ cells/mL using a hemocytometer. The culture was then incubated at 25°C on a rotary shaker at 200 rpm for 16 h.

### 2.2 Fungal pathogens

The fungal pathogens *B. cinerea* and *P. expansum* were isolated from infected apples and pears and identified by DNA sequencing of the ITS region, and maintained on potato dextrose agar (PDA) at 4°C. To reactivate the culture and confirm its pathogenicity, each pathogen was inoculated into a wounded apple, and re-isolated onto PDA after infection was successfully confirmed. Spore suspensions of the fungal pathogens were prepared from 2-week PDA plates incubated at 25°C, with spore concentrations determined using a hemocytometer and adjusted to 10^4^ spores/mL with sterile distilled water before use.

### 2.3 Test fruit

Apples (Malus × domestica Borkh) were harvested when commercially ripe. Only fruits that were uniform in shape and size, and free from physical damage, were selected for the study. The apples were thoroughly inspected to ensure they were intact and free from decay. To sterilize the fruit surfaces, a 2% (v/v) sodium hypochlorite solution was applied for 2 min, followed by rinsing with tap water and air-drying. The sterilized apples were then used in biocontrol experiments.

### 2.4 Xylitol treatment of *Meyerozyma guilliermondii*

In order to investigate the effect of xylitol treatment (XT) on *M. guilliermondii*, yeast cultures were centrifuged at 8,000 × g for 3 min. The yeast cells were washed three times with sterile distilled water to remove residual growth medium (Liu et al., 2012b). The cleaned cells were then resuspended in 20 mL of fresh YPD medium, and xylitol was added to achieve final concentrations of 0.5 or 1 M. The cultures were incubated on a rotary shaker at 25°C, with shaking at 200 rpm for 3 h. The xylitol concentrations were selected based on preliminary experiments. Briefly, given that xylitol concentrations typically range from 0.3 to 3 M in market food applications, we initially confirmed the efficacy of a lower concentration (1 M) in pilot experiments. To assess the potential effectiveness of even lower concentrations, we halved the treatment concentration to 0.5 M for further analysis. A control group, referred to as the Non-xylitol (NX) group, underwent the same procedure without xylitol addition. After centrifugation and washing, the yeast cells from both XT (0.5 and 1 M) and NX groups were resuspended in water at a concentration of 1 × 10^7^ cells/mL for further analysis.

### 2.5 Effect of xylitol on the survival rate of *Meyerozyma guilliermondii*

Cell samples collected at 0, 10, 20, and 30 min after oxidative treatment were cultured on YPDA agar medium (YPD supplemented with 20 g of agar per liter). The medium was incubated at 25°C for 3 days, after which colony-forming units (CFUs) per medium were quantified. Cell viability was expressed as the percentage of CFUs following oxidative treatment, relative to the CFU count of total cells. Each treatment contained three replicates, and the experiment was repeated three times.

### 2.6 Intracellular ROS, mitochondrial membrane potential, and ATP determination

Intracellular ROS levels in yeast cells were determined using the oxidation-sensitive probe 2′,7′-dichlorodihydrofluorescein diacetate (H_2_DCFDA; Invitrogen, Eugene, OR, USA), as previously described (Liu et al., 2011). Samples of NX and XT yeast cells were exposed to 30 mM H_2_O_2_ for 10, 20, or 30 min, with pre-exposure samples designated as time 0. The yeast cells were washed with phosphate-buffered saline (PBS, pH 7.0) and resuspended in the same buffer with 25 μM H_2_DCFDA. The suspension was incubated in the dark at 30°C for 1 h, washed twice with PBS, and examined under an FV3000 confocal microscope (Olympus, Tokyo, Japan) using a 480 nm excitation and 520 nm emission filter. ROS-positive cells were quantified by randomly selecting 10 fields per slide (with a minimum of 200 cells per slide), and the percentage of fluorescent cells relative to the total count was determined. Each experiment included three biological replicates and was repeated three times.

Mitochondria damage and ATP levels analysis was according to previous studies (Liu et al., 2012a; Sui and Liu, 2014). Briefly, mitochondrial membrane potential was assessed using the Mitochondrial membrane potential assay kit with JC-1 (Beyotime, China), which contains the cationic dye JC-1 (5,5′,6,6′-tetrachloro-1,1′,3,3′-tetraethyl-imidacarbocyanine iodide). This dye emits red fluorescence in the mitochondria of healthy cells. Upon collapse of the mitochondrial membrane potential, the cationic dye accumulates in the cytoplasm, emitting green fluorescence. Thus, the ratio of red to green fluorescence is higher in healthy cells and lower in damaged cells. In this study, all *M. guilliermondii* samples were collected and resuspended in the JC-1 reagent at a final concentration of 1 × 10^6^ cells/ml and incubated at 37°C for 15 min. Cells were then centrifuged and resuspended in 1 mL of assay buffer provided by the kit, and the ratio of red (excitation at 550 nm, emission at 600 nm) to green (excitation at 485 nm, emission at 535 nm) fluorescence was immediately measured using a Laser Scanning Confocal Microscopy (Zeiss, Germany). Each treatment contained three replicates, and the experiment was repeated three times.

ATP levels analysis was performed according to Li et al. (2010). Briefly, ATP of approximately 20 mg of fresh weight cultured *M. guilliermondii* cells that exposed to 30 mM H_2_O_2_ for 0, 10, 20, 30, 60, and 120 min were extracted with 50 μL of 2.5% trichloroacetic acid (TCA) and incubated at 4°C for 3 h. After centrifugation at 10,000 × g for 15 min, 10 μL supernatant was diluted with 115 μL ATP-free water and 125 μL ATP-free Tris-acetate buffer (40 mM, pH 8.0). ATP content was quantified using a luciferin/luciferase assay kit (Beyotime, China) according to the manufacturer’s instructions. Luminescence emission was measured using a Laser Scanning Confocal Microscopy (Zeiss, Germany). Each treatment contained three replicates, and the experiment was repeated three times.

### 2.7 Intracellular xylitol concentration measurement

To analyze the intracellular xylitol levels, yeast cells were disrupted by physical methods. After centrifugation at 8000 rpm at −4°C for 5 min, the yeast cell samples were collected and resuspended in HPLC-grade water. An HPLC system was employed to determine the xylitol concentrations (Agilent Series 1260, Agilent Technologies, Santa Clara, CA, United States). The mobile phase consisted of acetonitrile-water (80:20, v/v) with a flow rate of 1 mL/min. Xylitol was quantified using a standard (Sigma-Aldrich, Shanghai, China) with a linear response range of 0.05–10 mg/mL (Krallish et al., 1997). Xylitol concentrations of yeast cells were expressed as mg/g. Each treatment contained three replicates, and the experiment was repeated three times.

### 2.8 RNA isolation and reverse transcription–quantitative real-time PCR analysis of gene expression

Total RNA from NX and XT cells was extracted, treated with DNase, and purified using the EasyPure® Plant RNA Kit (TransGen Biotech, Beijing, China). RNA quality was assessed by gel electrophoresis and spectrophotometry (Nanodrop, Thermo Fisher Scientific, Waltham, MA, United States). First-strand cDNA was synthesized using 1 μg of total RNA, employing the TransScript® One-Step gDNA Removal and cDNA Synthesis SuperMix kit (TransGen Biotech). qPCR was performed on a Roche LightCycler® 480 (Roche, Basel, Switzerland) with the following program: 95°C for 5 min; 40 cycles of 95°C for 5 s and 60°C for 20 s; 95°C for 15 s; 60°C for 1 min, followed by a dissociation step at 95°C for 15 s. Gene expression of *TrxR* and *POD* was normalized to the reference gene *18S* rRNA using the 2^−ΔΔCT^ method (Livak and Schmittgen, 2001). Three independent biological replicates and three technical replicates were used, and the analysis was repeated three times.

### 2.9 Assay of enzyme activity

To measure antioxidant enzyme activity, XT and NX yeast cells were exposed to 30 mM H_2_O_2_ for 10, 20, or 30 min, with samples collected prior to oxidative stress exposure serving as time 0. Enzyme extracts were prepared according to the manufacturer’s instructions. Cells (1 × 10^8^) were frozen in liquid nitrogen, resuspended in chilled potassium phosphate buffer (0.1 M, pH 7.4), and centrifuged at 10,000 × g for 20 min at 4°C. The supernatant was collected for enzyme activity analysis. Activities of TrxR and POD were measured using commercial assay kits (Nanjing Jiancheng Bioengineering Institute, Nanjing, China) and expressed as U/mg protein. Protein content was determined using the Bradford assay, with bovine serum albumin as the standard (Bradford, 1976). One unit of TrxR activity was defined as the amount of enzyme that reduces 1 nmol of 5,5′-dithiobis-(2-nitrobenzoic acid) (DTNB) per minute at 25°C. One unit of POD activity was defined as the amount of enzyme that causes a 0.01 absorbance change per minute at 470 nm due to guaiacol oxidation. Each assay was performed with three biological replicates and repeated three times.

### 2.10 Biocontrol assay

The biocontrol efficacy was evaluated following the method outlined by Wang et al. (2018), with some adjustments. In brief, three wounds (4 mm deep × 3 mm wide) were created on the equator of each apple using a sterile nail. A 10 μL suspension (1 × 10^7^ cells/mL) of either XT or NX *M. guilliermondii* yeast cells was applied to each wound. One control group received sterile distilled water, while another control consisted of a 10 μL suspension (1 × 10^7^ cells/mL) of fresh yeast cells, which had not been exposed to sugar or oxidative stresses. The treated apples were divided into two groups. After the fruits were air-dried for 2 h, 10 μL of a *B. cinerea* or *P. expansum* suspension (1 × 10^4^ spores/mL) was introduced into each wound of the respective groups. The apples were then placed in covered plastic food trays, each sealed within a polyethylene bag, and stored at 25°C. After 4 days, the disease incidence and lesion diameter on each apple were measured. Incidence referred to the percentage of infected wounds, while lesion diameter was measured only for those wounds that showed infection. Each treatment was repeated three times with three replicates, each containing 10 apples. After 4 days treatments, uniformed apples of each treatment were photographed by a digital camera.

### 2.11 Data analysis

Statistical analyses were carried out using SPSS V26 (IBM Corp., Armonk, NY, United States). Comparisons between the NX and XT groups were made using Student’s t-test, with a significance level of *p* < 0.05. The results presented are based on pooled data from three independent experimental replicates, as a one-way analysis of variance (ANOVA) showed no significant effects of the treatments or interactions between variables and experiments.

## 3 Results

### 3.1 The effects of xylitol on the survival rate of *Meyerozyma guilliermondii* under oxidative stress

**Figure 1:**
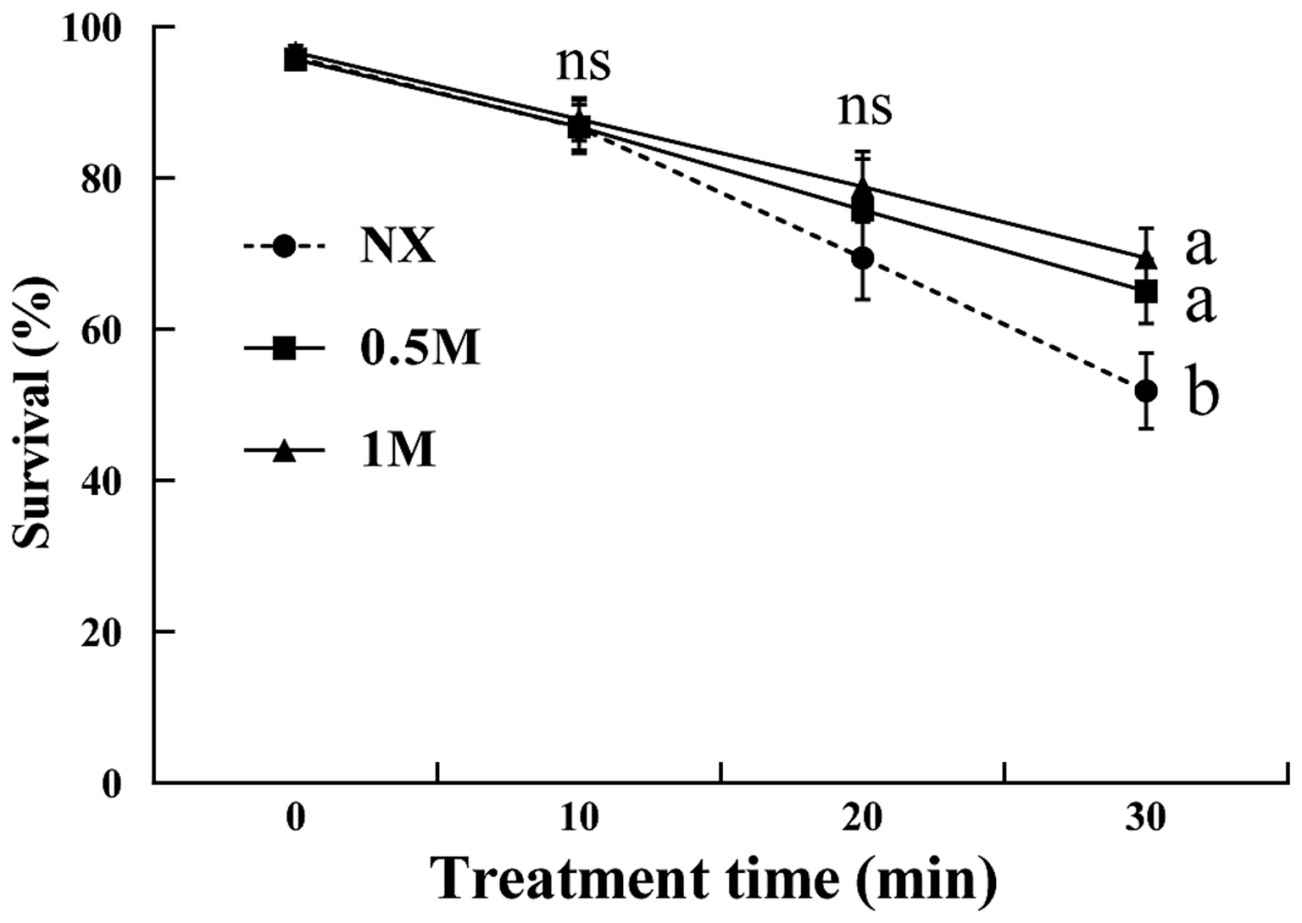
Percent viability of *Meyerozyma guilliermondii* cells under H_2_O or xylitol subjected to a subsequent oxidative (30 mM H_2_O_2_) stress for 10, 20 or 30 min. Prior to exposure to the subsequent oxidative stress served as Time 0. Data represent the mean ± standard deviation of three independent experiments, where each experiment consisted of three biological replicates (*n* = 9). Columns with different letters are significantly different according to a Duncan’s multiple range test at *p* < 0.05. ns, no significance.

Exposure to 30 mM H_2_O_2_ resulted in a decrease in cell viability as the treatment duration increased from 10 to 30 min (Figure 1). After 10 min of H_2_O_2_ exposure, the survival rates across all treatments were comparable, averaging around 86%. For yeast cells suspended in the NX group, survival rates dropped to 69 and 51% after 20 and 30 min of H_2_O_2_ exposure, respectively. Interestingly, survival rate of XT cells showed an increasing trend. Yeast cells treated with 1 M xylitol exhibited survival rates of 75 and 78% after 20 and 30 min of H_2_O_2_ exposure, respectively. Similarly, cells treated with 0.5 M xylitol exhibited over 65% viability under oxidative stress at both 20 and 30 min.

### 3.2 Effect of xylitol on cell damage and ATP levels in *Meyerozyma guilliermondii* under oxidative stress

**Figure 2:**
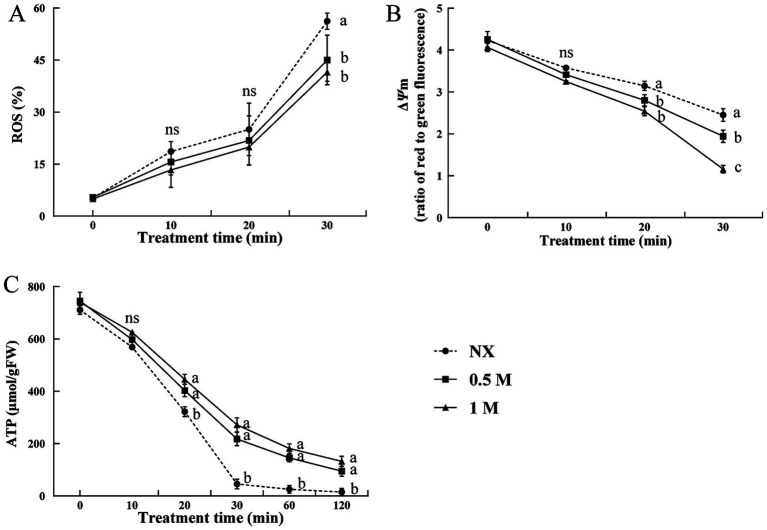
ROS accumulation **(A)**, mitochondrial membrane potential analysis **(B)** and ATP levels **(C)** of *Meyerozyma guilliermondii* cells under H_2_O or xylitol subjected to a subsequent oxidative (30 mM H_2_O_2_) stress for 10, 20 or 30 min, (60 and 120 min for ATP analysis). Prior to exposure to the subsequent oxidative stress served as Time 0. Data represent the mean ± standard deviation of three independent experiments, where each experiment consisted of three biological replicates (*n* = 9). Columns with different letters are significantly different according to a Duncan’s multiple range test at *p* < 0.05. ns, no significance.

At the time 0 (immediately following a 30-min pre-treatment but before exposure to oxidative stress), about 5% of cells treated with 0.5 M and 1 M xylitol, as well as the NX cells, were ROS-positive (Figure 2A). This proportion increased after exposure to 30 mM H_2_O_2_. At each time point, the cells treated with 0.5 M and 1 M xylitol showed significantly lower ROS levels compared to the NX cells. To investigate if the increase in ROS under oxidative stress was linked to mitochondrial dysfunction, Δ*Ψ*m and ATP content were measured. Figure 2B showed that Δ*Ψ*m was significantly changed between NX and XT cells under oxidative stress. And a decreasing trend of Δ*Ψ*m was observed in the XT cells compared to the NX group. As higher damage was observed in NX cells, ATP levels, reached their lowest point at 120 min when exposed to 30 mM H_2_O_2_ stress. In contrast, the ATP decline in XT cells was notably slower, cells treated with 1 M xylitol maintaining ATP levels approximately 10 times higher than those in the NX group at the 120-min (Figure 2C).

### 3.3 Yeast cells uptake xylitol under oxidative stresses

**Figure 3:**
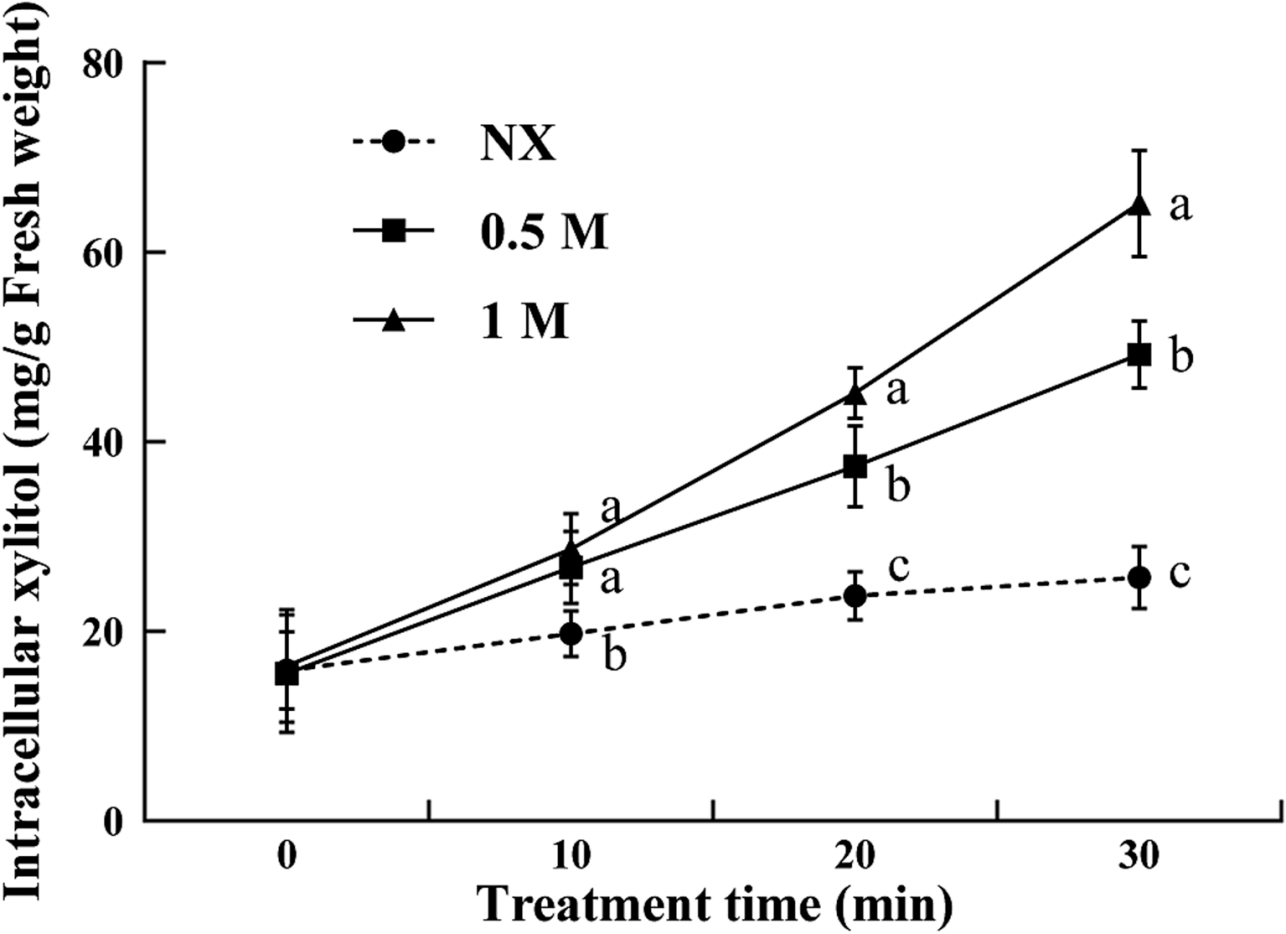
Intracellular xylitol concentrations of *Meyerozyma guilliermondii* cells under H_2_O or xylitol subjected to a subsequent oxidative (30 mM H_2_O_2_) stress for 10, 20 or 30 min. Prior to exposure to the subsequent oxidative stress served as Time 0. Xylitol concentrations represent as milligram per gram of yeast cell fresh weight (mg/g FW). Data represent the mean ± standard deviation of three independent experiments, where each experiment consisted of three biological replicates (*n* = 9). Columns with different letters are significantly different according to a Duncan’s multiple range test at *p* < 0.05. ns, no significance.

Compared to the NX group, yeast cells under oxidative stress exhibited a significant increase in intracellular xylitol levels following xylitol treatment. The intracellular xylitol concentration showed a positive relation to the increasing external xylitol concentrations. After 10 min treatment, the xylitol concentration in 0.5 and 1 M XT cells was significantly increased by 35.5% and 45%, respectively. When treated to 30 min, the highest intracellular xylitol levels were observed in 1 M XT cells (Figure 3).

### 3.4 Effect of xylitol treatment on the antioxidant system of *Meyerozyma guilliermondii*

**Figure 4:**
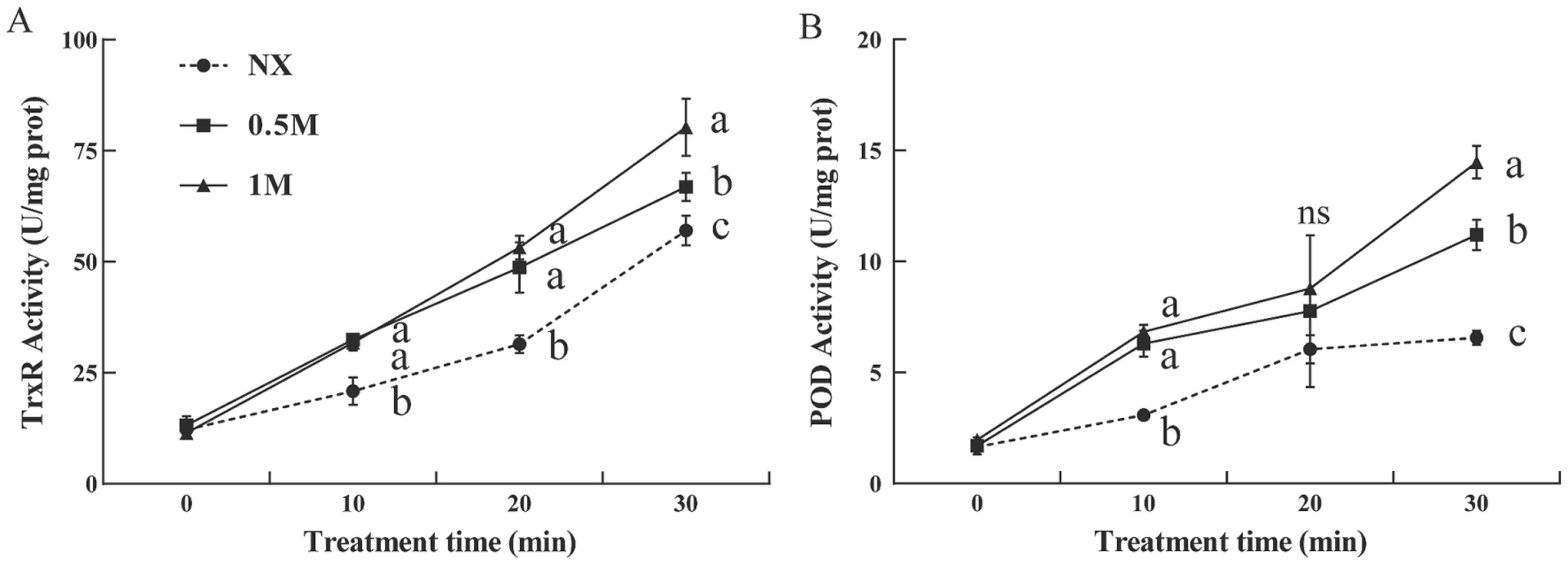
Thioredoxin reductase [**(A),** TrxR] and peroxidase [**(B),** POD] activity of *Meyerozyma guilliermondii* cells under H_2_O or xylitol subjected to a subsequent oxidative (30 mM H_2_O_2_) stress for 10, 20 or 30 min. Prior to exposure to the subsequent oxidative stress served as Time 0. Data represent the mean ± standard deviation of three independent experiments, where each experiment consisted of three biological replicates (*n* = 9). Columns with different letters are significantly different according to a Duncan’s multiple range test at *p* < 0.05. ns, no significance.

**Figure 5:**
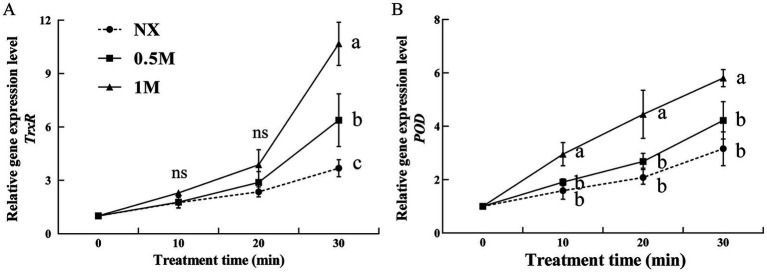
Two antioxidant genes [*TrxR* **(A)** and *POD* **(B)**] expression levels of *Meyerozyma guilliermondii* cells under H_2_O or xylitol subjected to a subsequent oxidative (30 mM H_2_O_2_) stress for 10, 20, or 30 min. Prior to exposure to the subsequent oxidative stress served as Time 0. Data represent the mean ± standard deviation of three independent experiments, where each experiment consisted of three biological replicates (*n* = 9). Columns with different letters are significantly different according to a Duncan’s multiple range test at *p* < 0.05. ns: no significance.

The activities of thioredoxin reductase (TrxR) and peroxidase (POD) were evaluated in pre-treated and NX samples in response to oxidative stress. The findings revealed that XT cells had significantly higher TrxR and POD activities compared to NX yeast cells (Figure 4). Under oxidative stress, TrxR activity in XT cells was significantly greater than in NX cells (Figure 4A). Similarly, POD activity in XT cells exposed to oxidative stress remained consistently higher than in NX cells across all time points (Figure 4B). In *M. guilliermondii* cells treated with xylitol, both the activities of these antioxidant enzymes and their corresponding gene expression levels (Figure 5) were significantly elevated at all-time points (from 0 to 30 min), both before and after exposure to oxidative stress, compared to untreated cells.

### 3.5 Biocontrol of postharvest diseases in apples using *Meyerozyma guilliermondii*

**Figure 6:**
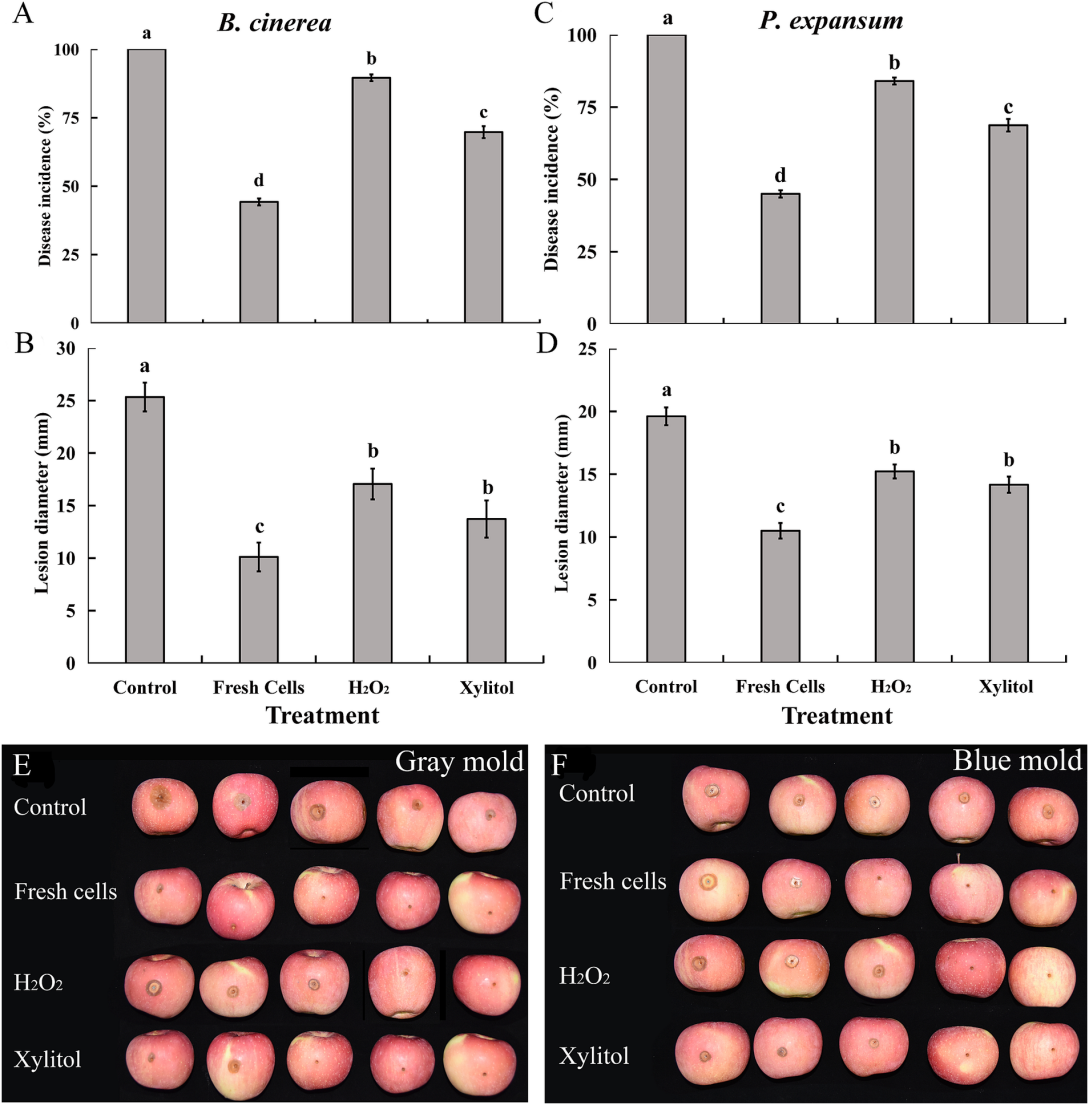
Biocontrol efficacy of *Meyerozyma guilliermondii* against gray mold caused by *Botrytis cinerea* **(A,B)**, blue mold caused by *Penicillium expansum* **(C,D)** in apples. Panels **(E,F)** showing the efficacy of xylitol on *M. guilliermondii* against gray mold and blue mold. Data represent the mean ± standard deviation of three independent experiments, where each experiment consisted of three biological replicates (*n* = 9). Columns with different letters are significantly different according to a Duncan’s multiple range test at *p* < 0.05.

As shown, the antagonistic yeast *M. guilliermondii* significantly reduced the incidence and lesion diameter of blue mold and gray mold, caused by *Botrytis cinerea* and *Penicillium expansum*, respectively, in apples. Notably, the incidence of both molds in fruit treated with fresh yeast was approximately 50% lower than in the controls, where disease incidence reached 100% for both pathogens. In contrast, the incidence of apple rot was significantly reduced in XT cells compared to the controls and H_2_O_2_ treatments (Figures 6A,C,E). Additionally, the lesion diameter on apples infected with *B. cinerea* and *P. expansum* was significantly smaller in fruit treated with XT cells than in the control and H_2_O_2_ groups (Figures 6B,D,F). These results demonstrate that *M. guilliermondii* effectively reduced the incidence and severity of blue mold and gray mold pathogens, with XT yeast cells further enhancing the level of control over the control treatments.

## 4 Discussion

Biocontrol agents used in postharvest disease management face challenges posed by various abiotic stresses in packing houses and postharvest environments, such as oxidative stress, elevated temperatures, nutrient deficiencies, and unfavorable pH levels. The ability of yeast to survive and remain active under these stresses is critical for improving their biocontrol efficacy (Wang et al., 2010; An et al., 2012). Since biocontrol yeasts are particularly vulnerable to oxidative conditions, their survival is significantly compromised under oxidative stress (Wang et al., 2018).

Xylitol, a sugar alcohol commonly used as a food additive, acts as an antioxidant, presenting a potential strategy to enhance the stress tolerance of yeasts (Kulikova-Borovikova et al., 2018; Wei et al., 2022). This study assessed the survival of yeast cultures treated with xylitol for 1 h under oxidative stress. The results demonstrate that xylitol treatment showed a significant promoting effect on the survival rate of *M. guilliermondii* under oxidative stress, with higher xylitol concentrations leading to a more pronounced protective effect. The XT yeast cells exhibited higher survival rates, may be attributed to two potential mechanisms. Firstly, the xylitol treatment provides limited osmotic protection, which reduced oxidative stress-induced damage (Karlgren et al., 2005). Secondly, the uptake of xylitol by yeast cells (Figure 3) may improve intracellular water utilization, thereby contributing to the resistance against oxidative stress (Krallish et al., 1997). It should be noted that the viability in this study was quantified as CFU, which may result in an underestimation of the actual level of viable cells. This is due to the fact that, following treatment, some yeast cells may have reduced vitality, preventing them from growing on the culture medium. However, these cells still exhibited detectable changes in enzyme activity and lower levels of ATP.

Previous research has demonstrated that exposure to oxidative stress can severely impair cell viability by increasing ROS production (Liu et al., 2011; Sui and Liu, 2014). The application of modified minimal mineral media, sugars, and sugar alcohols has been shown to mitigate the impact of high-temperature and oxidative stress in biocontrol yeasts by reducing intracellular ROS levels and minimizing oxidative damage (Sui and Liu, 2014; Ming et al., 2020). In this study, yeast cells treated with xylitol (XT group) exhibited lower levels of ROS, mitochondria dysfunction and higher ATP levels compared to the control cells. The correlation between lower ROS production, mitochondrial membrane potential and improved cell viability under oxidative stress suggests that xylitol enhances the oxidative resistance of yeast used as biocontrol agents. Since xylitol is involved in the glycolysis and the pentose phosphate pathway (Bertels et al., 2021; Narisetty et al., 2022), the uptake of xylitol by the yeast may regulate sugar metabolism or act as energy source, thus maintaining ATP production (Figure 3).

Antioxidant gene expression in yeast is typically upregulated in response to various stressors, helping the cells cope with environmental challenges (Martínez-Pastor et al., 2010; Huang et al., 2021). It has been reported that *POD* and *TrxR* participate in the oxidative response and enzymatic detoxification of ROS (Wang et al., 2018; Sun et al., 2021). In this study, xylitol treatment resulted in increased expression of *TrxR* and *POD* in yeast cells exposed to oxidative stress, which may be effective in enhancing the antioxidative defense of yeasts. Moreover, the enzyme activities of TrxR and POD, both essential for ROS detoxification (Yan et al., 2018; Sun et al., 2021; Huang et al., 2022), were significantly higher in XT cells. TrxR catalyzes the reduction of thioredoxin, while POD protects cells from ROS damage, playing a key role in biocontrol yeast (Greetham and Grant, 2009; Gostimskaya and Grant, 2016; Sun et al., 2021). The enhanced activity of these enzymes, along with their increased gene expression, indicated improved ROS clearance and higher survival rates in the XT yeast cells. Oxidative stress has also been shown to cause mitochondrial damage (Sui and Liu, 2014; Ming et al., 2020). In this study, oxidative stress significantly decreased ATP levels in the control cells, whereas xylitol treatment effectively mitigated this decline. The increase in survival, enhanced antioxidant enzyme activity, and reduction in ROS levels with xylitol treatment suggest a potential improvement in the biocontrol efficiency of yeast.

Xylitol, a widely utilized food additive, has been confirmed safe and healthy for consumption (Chen et al., 2010; Rice et al., 2020). Previous studies have indicated that under dehydration stress, yeasts such as *Saccharomyces cerevisiae* and *Pachysolen tannophilus* preferentially synthesize intracellular xylitol, which enhances their resistance to dehydration, with survival rates increased to 68% and 57%, respectively (Krallish et al., 1997). These findings underscore the potential of xylitol in enhancing yeast stress tolerance. For instance, treatment with 1 M xylitol elevated the viability of dried *S. cerevisiae* cells to 70% under dehydration, while a 2% (approximately 0.13 M) xylitol treatment improved the survival rate of *Zygosaccharomyces rouxii* from 65% to 69% following a 20-min exposure to 40°C heat stress (Kulikova-Borovikova et al., 2018; Wei et al., 2022). In this study, we systematically examined the impact of xylitol treatment on biocontrol yeasts under oxidative stress. Given xylitol’s involvement in the xylose metabolic pathway (Kumar et al., 2022), its absorption by yeast may enhance metabolic activity, thereby improving cell survival. Under oxidative stress induced by 30 mM H_2_O_2_, XT cells exhibited at least a 6% higher viability compared to previous reports. Furthermore, we demonstrated for the first time that xylitol treatment significantly mitigated oxidative damage in *M. guilliermondii*, with TrxR and POD activities increasing by at least 18 and 40%, respectively, leading to a 25% increase in ATP production. Notably, XT cells showed enhanced efficacy in reducing apple infection by *B. cinerea* and *P. expansum*.

High survival and growth of biocontrol yeasts on wounds and fruit surfaces provide them with a competitive edge in acquiring nutrients and space (Liu et al., 2013). XT yeast cells exhibited improved survival and biocontrol efficiency compared to untreated cells. Previous studies have also shown that pretreatment with protectants such as glucose and sorbitol can enhance the biocontrol efficacy of yeast (Sui et al., 2012; Sui and Liu, 2014; Ming et al., 2020). Lesion diameters were measured at their largest point, providing an indicator of the maximum extent of infection. Additionally, XT cells were more effective in reducing the incidence of gray mold and blue mold than the control, consistent with previous research, which demonstrated that pretreatments can improve stress tolerance and activate antioxidant defenses, leading to enhanced biocontrol efficiency (Liu et al., 2011; Chi et al., 2015).

## 5 Conclusion

This study demonstrated that xylitol treatment significantly enhances the tolerance and efficacy of *M. guilliermondii* under oxidative stress conditions. Xylitol treatment notably improved yeast survival rates, antioxidant enzyme activities and intracellular xylitol levels, thereby reducing the intracellular accumulation of ROS, mitochondrial dysfunction and preserving ATP levels during oxidative stress. The enhanced oxidative stress tolerance conferred by xylitol also contributed to improved biocontrol performance, as evidenced by reduced disease incidence and lesion diameter in apples subjected to pathogenic fungal infections. These findings underscore the potential of xylitol as an effective treatment to enhance the efficiency of biocontrol yeasts.

## Data Availability

The original contributions presented in the study are included in the article/supplementary material, further inquiries can be directed to the corresponding author.
